# Analysis of chemical compositions and larvicidal activity of nut extracts from *Areca catechu* Linn against *Aedes* (Diptera: Culicidae)

**DOI:** 10.1371/journal.pone.0260281

**Published:** 2021-11-29

**Authors:** Madhuri Bharathithasan, Darvin R. Ravindran, Dinesh Rajendran, Sim Ka Chun, S. A. Abbas, Sandheep Sugathan, Zary Shariman Yahaya, Abd Rahman Said, Wen-Da Oh, Vijay Kotra, Allan Mathews, Mohamad Faiz Mohd Amin, Intan H. Ishak, Rajiv Ravi

**Affiliations:** 1 School of Applied Sciences, Faculty of Integrated Life Science, Quest International University, Ipoh, Perak, Malaysia; 2 Faculty of Medicine, Quest International University, Ipoh, Perak, Malaysia; 3 Insecticide Resistance Research Group (IRRG), School of Biological Sciences, Universiti Sains Malaysia, Minden, Penang, Malaysia; 4 Vector Control Research Unit, School of Biological Sciences, Universiti Sains Malaysia, Minden, Penang, Malaysia; 5 Faculty of Pharmacy, Quest International University, Ipoh, Perak, Malaysia; 6 School of Chemical Sciences, Universiti Sains Malaysia, Penang, Malaysia; 7 Faculty of Earth Science, Universiti Malaysia Kelantan, Jeli, Kelantan, Malaysia; Banaras Hindu University, INDIA

## Abstract

**Background:**

There is a growing need to use green alternative larvicidal control for *Aedes* larvae compared to chemical insecticides. Substantial reliance on chemical insecticides caused insecticide resistance in mosquito populations. Thus, research for alternate chemical compounds from natural products is necessary to control *Aedes* larvae. This study explores the analysis of chemical compositions from *Areca catechu* nut as a potential larvicide for *Aedes* (Diptera: Culicidae).

**Methods:**

The *Areca catechu* nut collected from Ipoh, Perak, Malaysia was grounded into powder and used for Soxhlet extraction. The chemical analysis of the extracts and their structures were identified using the GCMS-QP2010 Ultra (Shimadzu) system. National Institute of Standards and Technology (NIST) Chemistry WebBook, Standard Reference Database 69 (https://webbook.nist.gov/chemistry/) and PubChem (https://pubchem.ncbi.nlm.nih.gov/), the two databases used to retrieve the synonyms, molecular formula, molecular weight, and 2-dimensional (2D) structure of chemical compounds. Next, following WHO procedures for larval bioassays, the extracts were used to asses larvicidal activity against early 4^th^ instar larvae of *Aedes aegypti* and *Aedes albopictus*.

**Results:**

The larvicidal activities were observed against early 4^th^ stage larvae with different concentrations in the range from 200 mg/L to 1600 mg/L. The LC_50_ and LC_95_ of *Aedes aegypti* were 621 mg/L and 2264 mg/L respectively; whereas the LC_50_ and LC_95_ of *Aedes albopictus* were 636 mg/L and 2268 mg/L respectively. Mortality was not observed in the non-target organism test. The analysis using gas chromatography and mass spectrometer recovered several chemical compounds such as Arecaidine, Dodecanoic acid, Methyl tetradecanoate, Tetradecanoic acid <n->, and n-Hexadecanoic acid bioactive components. These chemical constituents were used as additive formulations in pesticides, pest control, insect repellent, and insecticidal agents.

**Conclusions:**

Our study showed significant outcomes from the extract of *Areca catechu* nut and it deserves further investigation in relation to chemical components and larvicidal actions between different species of *Aedes* mosquitoes. Even though all these findings are fundamental, it may have some interesting potentials to be developed as natural bio-larvicidal products.

## Introduction

Mosquitoes became major vector-borne diseases such as dengue, malaria, yellow fever, Zika, chikungunya, and lymphatic filariasis. Dengue is a major disease transmitted by *Aedes* mosquitoes and it has been an unresolved problem in the tropical regions. During the Covid-19 pandemic period, based on dengue updates by World Health Organization [1], Malaysia had a total of 82,753 cases and 133 deaths in cumulative in 2020 and the Philippines had a total of 71,785 cases along with 277 deaths, cumulative from 1 January to 17 October 2020. Singapore had 32,494 cases which were recorded on 24 October 2020 and were the largest outbreak in the history of Singapore. Besides that, Vietnam had reported a total of 70,585 cases of dengue with 7 deaths during 2020.

The *Aedes aegypti* and *Aedes albopictus* are the major vectors of the dengue virus(DENV) [1]. Controlling vector-borne diseases is a daunting task, even though various methods had been used for many years. The most common methods of adulticidal and larvicidal measures in practice are chemical, physical and biological measures. Even with all these measures, mosquitoes had developed resistance against common insecticides such as Dichlorodiphenyltrichloroethane (DDT) as the first chemical used against adult mosquitoes followed by Malathion and Pyrethroids [2]. Biological control measures act as an alternative to chemical methods and it began with the introduction of fish to ingest larvae and the use of transgenic vectors to reduce the capacity of mosquito reproduction [3]. Mosquitoes have major advantages in developing resistance towards insecticides within a short period of time [4]. There are several types of common resistance found among mosquitoes such as target site resistance, metabolic resistance, behaviour resistance, and cuticle penetration resistance [4, 5].

After several studies on mosquito resistance issues, researchers have decided to work with bioinsecticides as an alternative approach [4, 5]. Bioinsecticides are derived from natural resources such as plant extracts having active chemical components from leaves, flowers, seeds, and roots. The *Areca catechu* is a potential plant to be used as a bioinsecticide due to its wide availability in tropical and subtropical regions. *Areca catechu* is also known as betel nut or areca palm. The nut of *A*. *catechu* can be applied as an additive material and also a stimulant [6]. Polyphenol, fatty acids, alkaloids, starch, flavanols, and other minerals are the few main phytochemical components from *A*. *catechu* nuts [7]. Previous studies reported that phytochemical components such as fatty acids and flavonoids can be the cause of high mortality rates on larvae of *Aedes*, *Anopheles*, and *Culex* mosquito species [8]. It has also been reported that the leaf extracts of *A*. *catechu* caused significant mortality towards *Aedes aegypti* larvae [9]. Until now, the effects of *A*. *catechu* nut extract on the larvicidal activity of *Ae*. *aegypti* are limited. There are also no related studies on the larvicidal effects of *A*. *catechu* nut extract on *Ae*. *albopictus*. Thus, further studies on *A*. *catechu* nut are essential as it may become a potential bio-insecticide. Following this concept, the objective of this study is to elucidate the analysis of chemical compounds from *A*. *catechu* nut extracts and their larvicidal action against the early 4th instar larvae of *Aedes aegypti* and *Aedes albopictus*.

## Methodology

All the experimental protocols in this paper were based on World Health Organization (WHO) larval susceptibility test method guidelines [10]. All experimental procedures were approved by animal ethics: USM/IACUC/2020/112/910 from Vector Control Research Unit, School of Biological Sciences, Universiti Sains Malaysia, Minden, Penang, Malaysia.

### Plant materials

**Fig 1:**
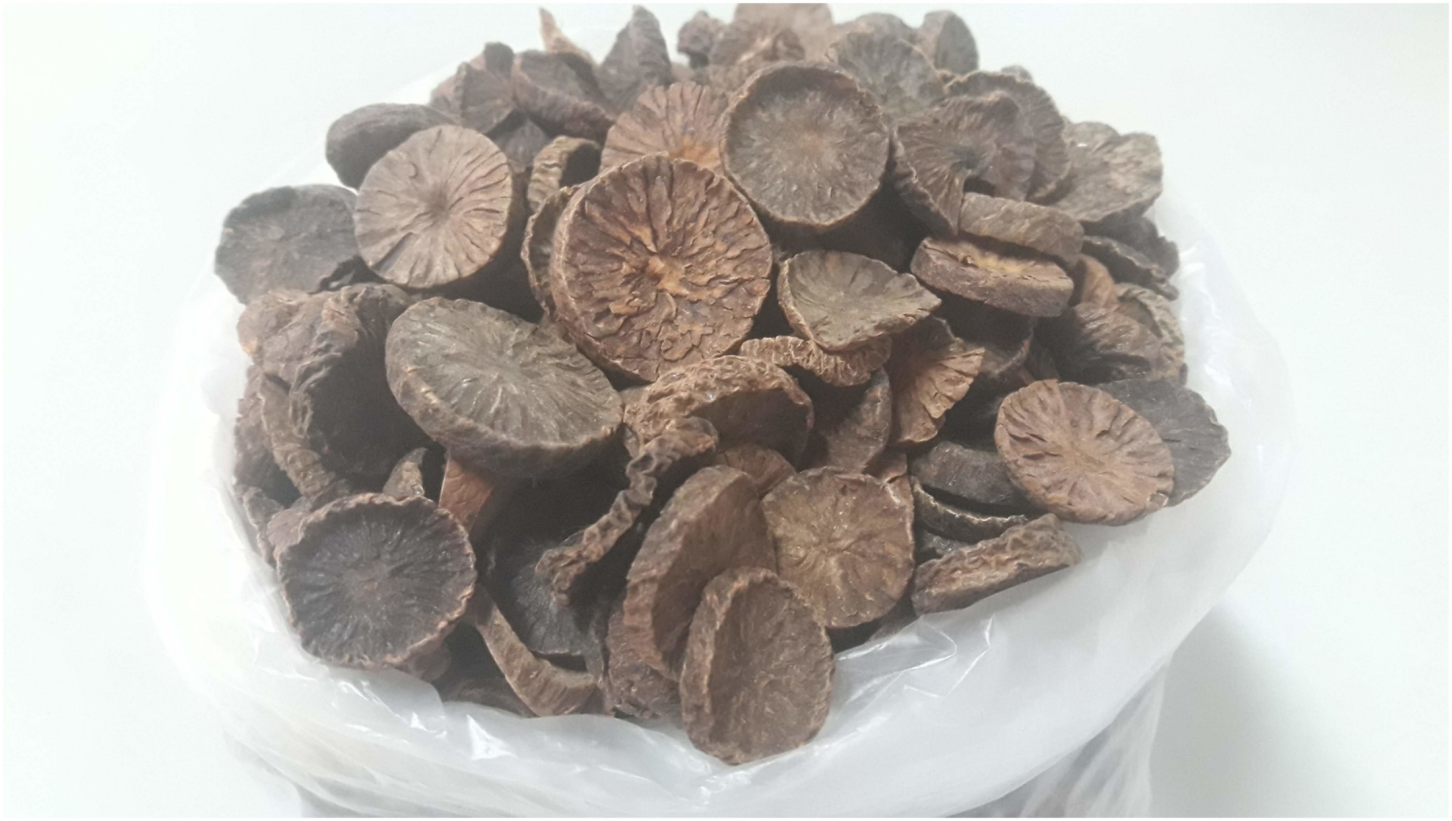
Picture of *Areca catechu* nut from the field. The ventral view of phyllotaxis from *Areca catechu* nut.

*Areca catechu* (Fig 1) was purchased commercially from a local vendor in Ipoh, Perak, Malaysia (4 42.979 N, 101 07.278 E). The nuts were washed in running tap water to remove debris and other foreign particles. Then it was dried under less than 40°C overnight in a hot air oven to dry excess water. *Areca catechu* was grounded into powder using an industrial grinder [11]. The powder was stored in a vacuum-tight container for future usage.

### Soxhlet extraction

The extraction was performed based on Soxhlet extraction protocols [12]. A total volume of 1000 ml absolute methanol was used to extract 40g of *A*. *catechu* powder and the extractions were concentrated using a rotary evaporator at a temperature of 45°C. Finally, the concentrated extract was stored in a universal bottle and maintained at a temperature of 4°C [12, 13].

### Larvae rearing

The eggs of *Aedes aegypti* and *Aedes albopictus* were obtained from Vector Control Research Unit (VRCU) at University Science Malaysia (USM) Penang, Malaysia [12, 14, 15]. These *Aedes* eggs were placed in dechlorinated water for 24 hours until they hatched. Emerged larvae were reared at separate plastic trays with not more than 200 larvae per tray [16]. The larvae rearing condition was maintained at a temperature of 28±2°C and relative humidity of 70–85%, photoperiod of 14:10 hours (light/dark) in the laboratory [12, 17, 18]. The early 4^th^ instar stages of mosquito larvae were used for bioassay test [12, 18].

### Larvicidal bioassay

The larvicidal bioassays test were conducted following World Health Organization guidelines,[10]. A total number of 100 forth instar *Aedes* larvae were separated into disposable plastic cups with 200 mL dechlorinated water for each concentration. Five replicates were used for each concentration with methanol (CH_3_OH) set as control [18, 19]. Furthermore, fish foods were provided to these larvae during the bioassays test [12]. The methanol control solutions were prepared by adding 1 mL of distilled water with 10% of methanol on every replicate [12, 17]. Total of 8 test concentrations were selected within the estimated test ranges of 100 mg/L to 1800 mg/L for the bioassays test, conducted in 3 separate days and named as Day 1, Day 2, and Day 3 [12, 18]. Following the World Health Organization Guidelines [10], the mortality of larvae for every concentration was observed and recorded within the period of 24 hours and 48 hours at a room temperature of 28 ± 2°C. Any absence of mobility from the larvae is considered as mortality [20].

### Morphological view

An optical microscope (Leica USM) with 40-400x magnification was used to view the condition of early 4^th^ instar larvae of *Aedes aegypti* and *Aedes albopictus* [21].

### Non-target organism test

Ten Guppy fishes (*Poecilia reticulata)* were used in three replicates. Groups were tested with the larvicidal lethal concentrations of LC_95_ of 3000 mg/L, 2700 mg/ L, and 2500 mg/L for 24 hours with frequent observation on abnormalities or mortality of the fishes [12, 18]. The methanol control solutions were prepared by adding 1 mL of distilled water with 10% of methanol on every replicate [12, 17]. The water condition was tested based on pH, temperature, and oxygen levels in the beginning and at the end of experiments, fish tank volume of 5L each set for each concentration. At the post-experiment, fishes were handled with mitigating pain, suffering, and distress by transferring into a holding fish tank with adequate aeration, constant water temperature for recovery monitoring for 24 hours before releasing it into the natural pond environment. Histological analysis of fish liver for all concentrations has been randomly selected according to protocols from a previous study by Vajargah et al. [22].

### GC—MS analysis

The GC—MS analysis for the crude extract was conducted according to Ravi et al. [12] and Ravindran et al. [18]. GCMS-QP2010 Ultra (Shimadzu) system was fitted with a RTX5 capillary column (30 m × 0.25 mm of internal diameter, × 0.25 μm of film thickness and maximum temperature of 370°C) and was paired to a QP2010 Ultra (Shimadzu) MS. Ultra-high purity helium (99.99%) was used as a carrier gas and 1.0 mL/ min was set as a constant flow rate. A temperature of 280°C was used for injection, transfer line, and ion source. The oven temperature was programmed at 80°C (hold for 2 min) to 280°C at a rate of 3°C/min. A suitable solvent (1/100, v/v) was used to dilute the crude extracts and it was also filtered. A syringe was used to take the diluted crude extract (1 μL) without any particles and it was injected into an injector with a split ratio of 10:1. The scan range of 40–550 amu in the full-scan mass spectra was collected as data for this study. The percentage composition of the crude extract constituents will be determined by the percentage of peak area. The chemical compounds of crude extracts from Soxhlet extraction were identified and characterized based on the GC retention time. The NIST 08 mass spectrum libraries standards were matched to compare the mass spectra.

### Analysis of chemical compounds of *Areca catechu* nut

National Institute of Standards and Technology (NIST) Chemistry WebBook, Standard Reference Database 69 (https://webbook.nist.gov/chemistry/) and PubChem (https://pubchem.ncbi.nlm.nih.gov/) were the two databases used to retrieve synonyms, molecular formula and molecular weight of the chemical compounds. PubChem (https://pubchem.ncbi.nlm.nih.gov/) and ChemSpider (http://www.chemspider.com/) were also used to retrieve some chemical and physical properties of chemical compounds such as colour, form, odour, boiling point, melting point, and solubility. Google Patent (https://patents.google.com/) was used to retrieve the patented lists on *A*. *catechu* nut for pesticide formulations.

### Statistical analysis

IBM SPSS Statistics 24 used to analyse data by probit analysis [18].

## Results

### Larvicidal bioassays

**Fig 2:**
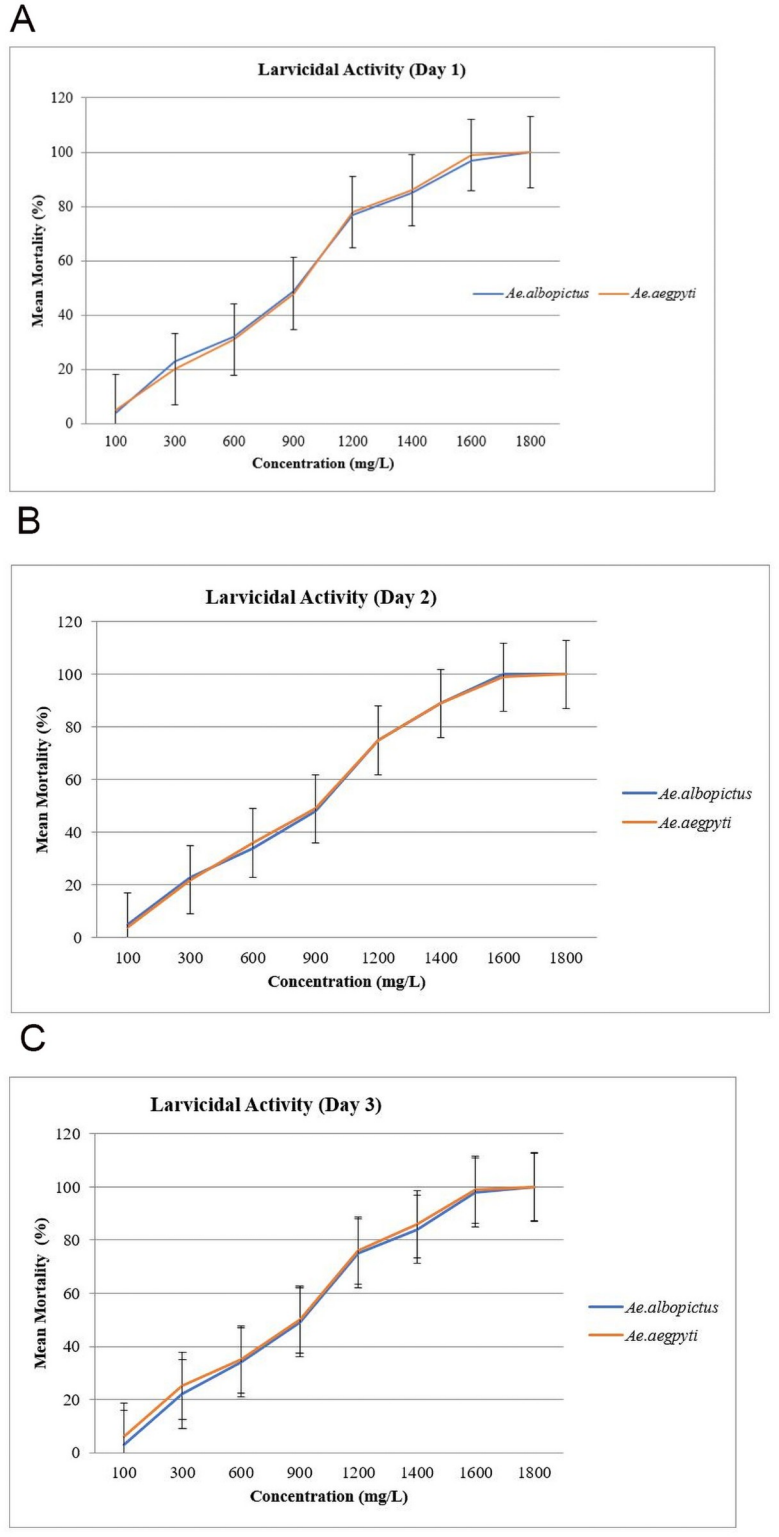
(A-C) Mortality rate from Day 1 till Day 3 of *Aedes aegypti* and *Aedes albopictus* larvae after exposure to *Areca catechu* nut extracts.

There was a significant increase in the mortality percentage with the increase in concentrations of *A*. *catechu* extracts. Based on the comparisons with *Aedes albopictus*, larvicidal activities were at much lower concentration on the 4^th^ instar larvae of *Aedes aegypti* with LC_50_ and LC_95_ values of 621 mg/L and 2264 mg/L, respectively (Table 1). The bioassays test for *Aedes albopictus* larvae was recorded at LC_50_ and LC_95_ values of 636 mg/L and 2268 mg/L, respectively (Table 1). The graphical representation of larval mortality rates from Day-1 to Day-3 for *Aedes aegypti* and *Aedes albopictus* were shown in Fig 2A, 2B and 2C. The LC_50_ and LC_95_ values with 95% confidence intervals (CI), chi-square, and degree of freedom (df) value were calculated and tabulated as in Table 1. Finally, there was no significant mortality in the control assays.

**Table 1:** Larvicidal activity of *A*. *catechu* extracts against early 4th instar larvae of *Aedes aegypti* and *Aedes albopictus*.

Larvae	Na	LC50(mg/L) (95% LCL-UCL)	LC95(mg/L) (95% LCL-UCL)	χ2	df	R
*Ae*.*aegpyti*	300	621	2264			
		(576–666)	(2022–2586)	36*	12	0.642
		Y = -8.185+2.930X	Y = -8.185+2.930X			
*Ae*.*albopictus*	300	636	2268			
		(592–679)	(2039–2569)	37*	13	0.659
		Y = -8.353+2.979X	Y = -8.353+2.979X			

Na: total number of mosquitoes larvae used, n = 25 each with 4 replicates for 3 days; LC_50_: lethal concentration for 50% mortality; LC_95_: lethal concentration for 95% mortality; LCL: lower confidence limits; UCL: upper confidence limits; χ2: Pearson chi-square; df: degrees of freedom; R: Pearson’s R (note: chi-square values with an asterisk are significant (P < 0.05).

### Morphological view

**Fig 3:**
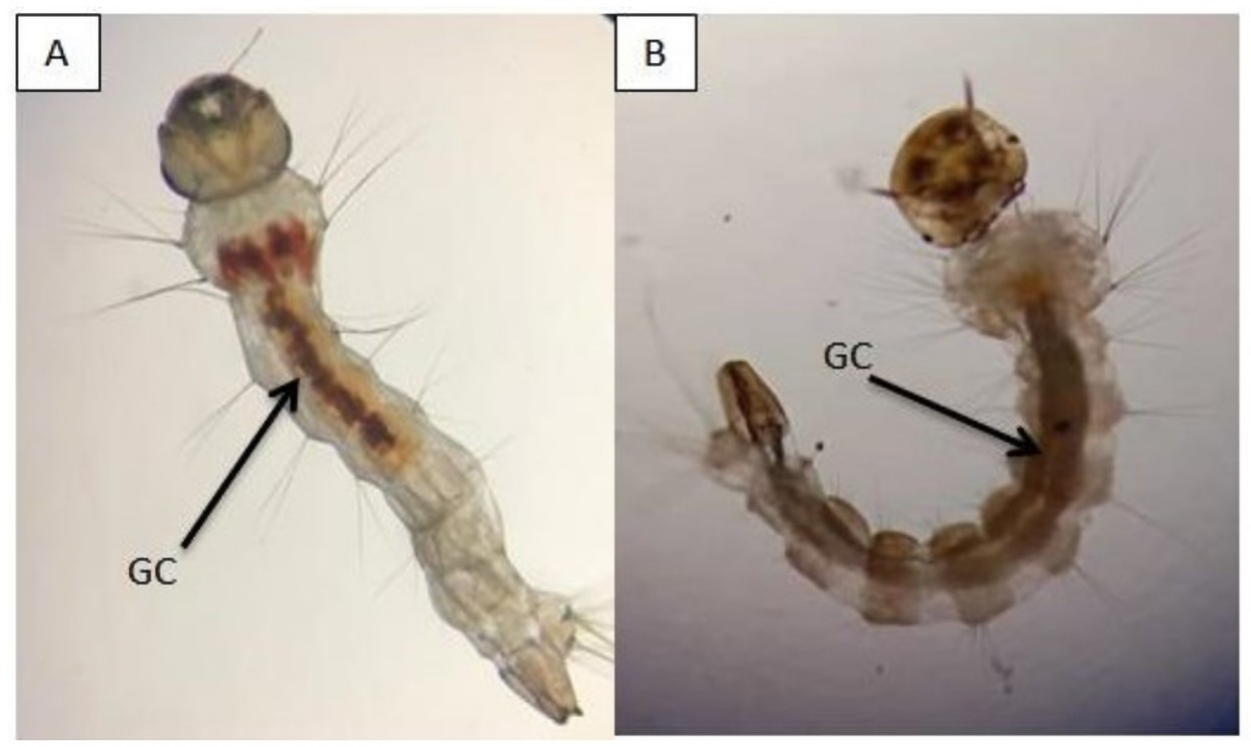
Morphology of *Aedes* mosquito midgut after exposure to *Areca catechu* nut extract. **(A) *Aedes aegypti* larvae. (B) *Aedes albopictus* larvae**. Note: Arrow indicates the plant extract (reddish-brown colour) present in the midgut; GC: gut content (after 24 hours).

Fig 3A and 3B showed the morphological view of larvae with the presence of *A*. *catechu* nut extracts inside the midgut regions.

### Non-target organisms test

**Fig 4:**
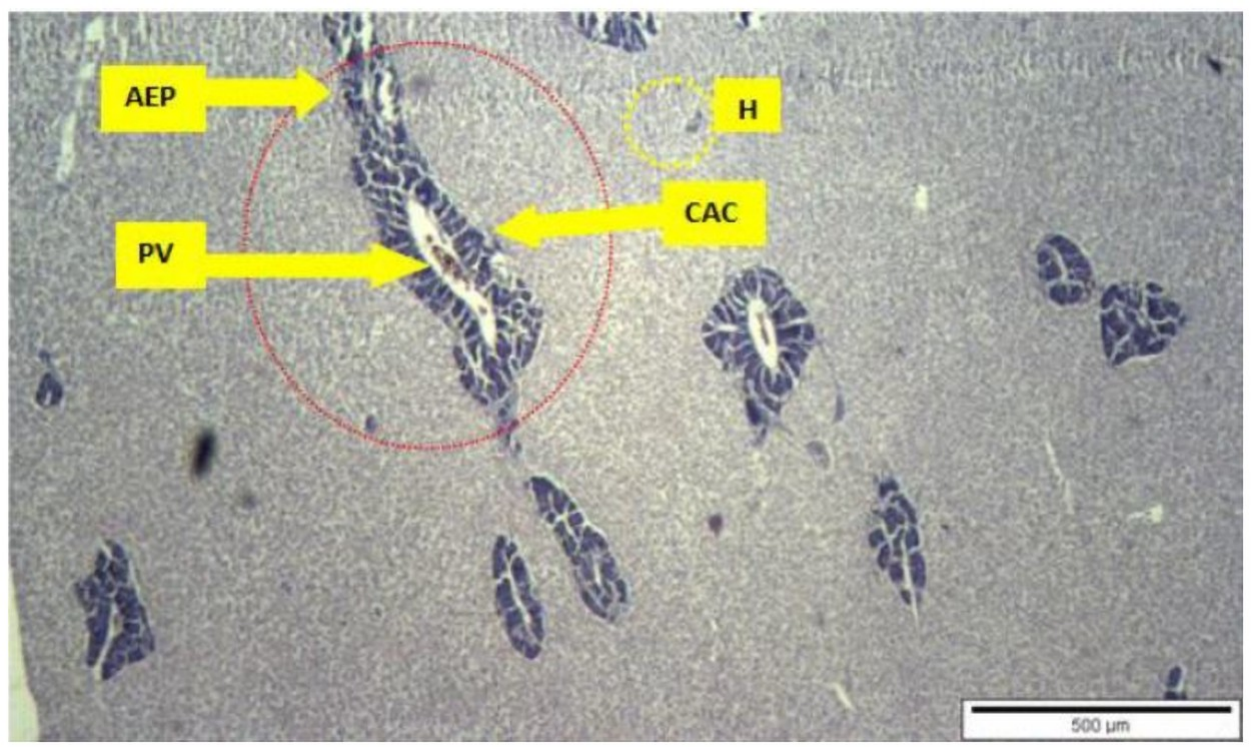
Liver stained with haematoxylin and eosin, bar 500μm, whereby all the concentrations of non-target organisms test showed undamaged hepatic architecture hepatocytes (H), acini exocrine pancreas (AEP) with centro acinar cells (CAC) and portal vein (PV).

The outcome of the non-targeted organism bioassay of guppy fish, *Poecilia reticulata*, showed no mortality with *A*. *catechu* extracts ranging from 2500 mg/L to 2700 mg/L but with the concentration of 3000 mg/L, it was 1% mortality rate after 24 hours observations. The outcome from 2700mg/L clearly showed no mortalities and was safe for the guppy fishes which comply with the concentration limit range for larvicidal activity in this study. However, if the concentration value for *A*. *catechu* nut extracts exceeds the point of 2700ppm till 3000ppm, there could be 1% mortality. Additionally, no mortalities were observed at all controls. Fish liver histology analysis from all concentrations, including the control test in this study showed no signs of damages in terms of necrosis and vacuolization (Fig 4).

### GC—MS analysis and chemical compound screening

**Fig 5:**
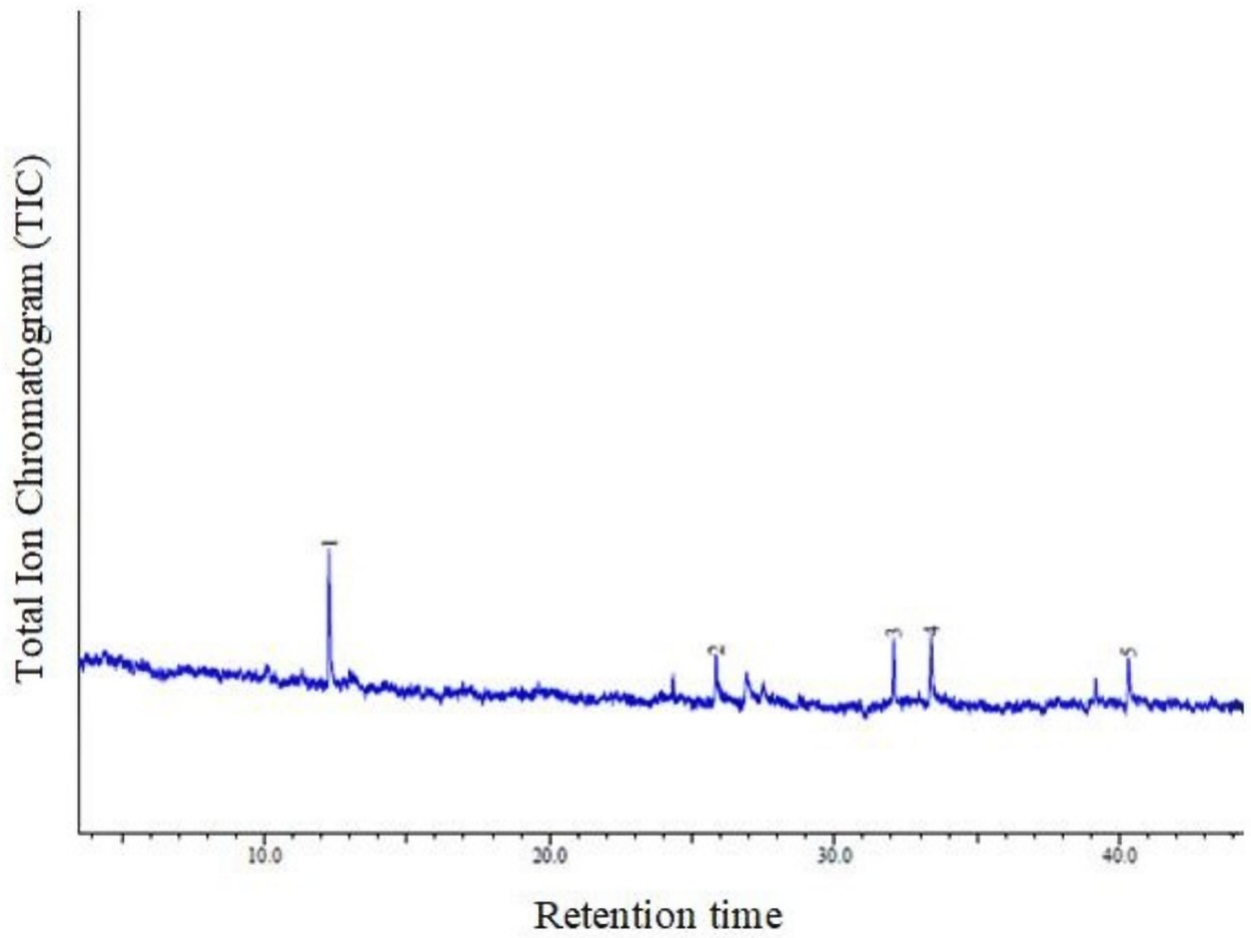
Chromatogram for GC-MS analysis for *Areca catechu* nut. (ACQUISITION PARAMETERS; RTX5 capillary column 30 m×0.25 mm inner diameter, ×0.25 μm film thickness, the oven temperature was programmed from 80°C (hold for 2 min) to 280°C at a rate of 3°C/min Carrier Gas = He).

The GC—MS analysis of *A*.*catechu* nut extracts showed five peaks and the presence of five different types of chemical compounds compared with NIST 08 library (Table 2, Fig 5). The components recorded were; Arecaidine, Dodecanoic acid, Methyl tetradecanoate, Tetradecanoic acid <n->, and n-Hexadecanoic acid. Supplementary file, S1 as attached for chemical compounds from NIST 08 library.

**Table 2:** Chemical compounds for *Areca catechu* nut.

S/N	RT	Area	Area (%)	Compound name	Activity
1	12.261	19923	31.281	Arecaidine	Anthelmintic[23]
2	25.848	8545	13.417	Dodecanoic acid	Antimicrobial[24]
3	32.083	19564	30.718	Methyl tetradecanoate	Nematicide[25]
4	33.397	7812	12.266	Tetradecanoic acid <n->	Larvicidal[26]
5	40.315	7846	12.319	n-Hexadecanoic acid	Larvicidal[26]

S/N: signal notice, RT: retention time

### Analysis of chemical compounds of *Areca catechu* nut

Basic chemical compound information of *A*. *catechu* nut and its potential utilization of chemical compounds for pesticide formulations were as shown in Table 3 and S1 Table.

**Table 3:** Potential utilization of chemical compounds within *A*. *catechu* nut for pesticide formulations.

Patent Name	Formulation Types	Chemical compounds	Patent Number
Synergistic pesticide composition	Adjuvant mixtures	Hexadecanoic acid methyl ester, oleic acid, palmitic acid, 9-octadecenoic acid (Z)-methyl ester, catechin	US9028856B2 [27]
Safer,organophosphorous compositions	Adjuvant mixtures	Hexadecanoic acid methyl ester, oleic acid, palmitic acid, 9-octadecenoic acid (Z)-methyl ester, tetradecanoic acid	US6566349B1 [28]
Pest control using natural pest control agent blends	Pesticide constituents	Oleic acid, palmitic acid, tetradecanoic acid	US10368543B2 [29]
Pesticidal compositions and methods of use thereof	Repellent or cidal agents	Oleic acid	US9756857B2 [30]

## Discussion

Current findings showed that bioactive compounds from *A*. *catechu* nut extracts have the potentials to be an alternative control measure for *Aedes* larvae, compared to synthetic insecticides. This is due to the high mortality recorded on *Ae*. *aegypti* and *Ae*. *albopictus* larvae during the exposure with *A*. *catechu* nut extracts. Current, study has screened five chemical compounds which are generally classified as antimicrobial and larvicidal agents.

The first chemical compound was Arecaidine, which is also known as arecoline hydrobromide. Arecoline hydrobromide is used as an anthelmintic in the process of treating tapeworm infections on dogs [31]. Next is dodecanoic acid which is screened from this study, can also be referred to as lauric acid, and is a saturated fatty acid with antimicrobial and insecticide properties [32, 33]. Another chemical compound which was screened is Methyl tetradecanoate, also known as tetradecanoic acid or myristic acid. This chemical compound has been related to fragrances and flavouring agents but also contains methyl tetradecanoate derivatives with larvicidal activities [34]. Finally, another compound which was screened was n-Hexadecanoic acid with a common name of palmitic acid with strong larvicidal effects which can be found similarly on other plants such as *Azolla pinnata* and *Clitoria ternatea* [12, 18].

A previous study with *A*. *catechu* nut by Mading et al. [35] showed that ethanol extraction with high concentrations showed mortalities in *Anopheles vagus* larvae. Similarly to recent studies by Ravindran [18] and Ravi et al. [36], *A*. *catechu* nut extract in this study had shown mortalities at 4^th^ instar larvae with ingested extracts(reddish-brown appearance) on the midgut of *Ae*. *aegpti* and *Ae*. *albopictus*; as shown in Fig 3A and 3B. Recent studies of Malaysian plants such as *Azolla pinnata* and *Clitoria ternatea* showed larvicidal efficacies for *Ae*. *aegpti* and *Ae*. *albopictus* between LC50 and LC95 values of 1000ppm till 2500ppm but in this study, a lower LC50 and LC95 values of 620ppm till 2300ppm were reported [12, 18, 36]. Thus, we can safely conclude that *A*. *catechu* nut may be the best candidate for natural bio-larvicidal applications in Malaysia. Besides that, according to a review by Silverio et. [37], there are various studies globally with lower LC50 and LC95 values on several plants, extraction solvents, and essential oils applicable against *Ae*. *aegpti* mosquito larvae. The vast differences in LC50 and LC95 values are due to plant locality, plant resources, extraction techniques and stability of plant based chemical components. Furthermore, *A*. *catechu* nut extract did not cause toxic effects to non-targeted organisms such as guppy fish. Based on the test performed the highest concentration used against larvae which were 2700 mg/L, did not bring toxic effect to the non-targeted organisms but by increasing the concentration to 3000 mg/L it had a mild effect on the fishes and caused a mortality rate of 1% within 24 hours. Similarly, to recent studies on non-target organisms test conducted by Ravindran [18] and Ravi et al. [36], the plant extracts in this current study also showed non-toxic effects. However, the application in field tests might escalate the lethal concentrations and may cause some toxicity towards guppy fishes and this deserves a future investigation during field test applications.

Larger components of chemical compounds from *A*. *catechu* nut were mostly from fatty acids, fatty acid methyl esters and flavonoids. All these chemical compounds had been included in various patents for pesticide formulations and applications. Both, Anderson et al. [28] and Reid et al. [27] had invented the constituents containing mixtures of various adjuvants within the composition of pesticides. Fatty acids (oleic acid, palmitic acid, tetradecanoic acid) and fatty acid methyl ester (hexadecanoic acid methyl ester, 9-octadecenoic acid (Z)-methyl ester), and flavanols (catechin) were also included in their inventions. According to Mirgorodskaya et al. [38], adjuvant plays its role in enhancing the penetration capacity of the bioactive component as insecticides. Additionally, Anderson et al. [28] and Reid et al. [27] also believed that these invented adjuvant mixtures can help to improve the penetration rates of insecticide within the cuticles of adult mosquitoes and during adulticidal bioassays test. These gives us some conceptions that, the same *A*. *catechu* nut extracts can be tested on various life stages of *Aedes* mosquitoes.

Another interesting finding, fatty acids such as oleic acid, palmitic acid, and tetradecanoic acid were also found within the oil of black seed *Nigella sativa*. These fatty acids were utilized in their invention to produce the synergistic pesticides constituents [29]. Besides that, Jones [30] invented pesticides which consisted of a combination of sodium lauryl sulfate and several types of fatty acids against insects. Oleic acid was one of the fatty acids which could be applied within this invention as a repellent agent. According to Bosch et al. [39] and Ali et al. [40], some of the fatty acids, especially saturated fatty acids had been reported as having repellent properties against *Aedes aegypti* mosquitoes. Based on the information on these patented inventions, all these fatty acids, fatty acid methyl esters, and flavanol are having the potential for the development of larvicidal products against *Aedes* mosquitoes using *A*. *catechu* nut extracts.

Some of the chemical components such as oleic acid, palmitic acid, and tetradecanoic acid were the fatty acids found within *A*. *catechu* nut. Some previous studies reported that the toxic properties of oleic acid (unsaturated fatty acids) towards *A*. *albopictus* larvae were higher compared to palmitic acid and tetradecanoic acid (saturated fatty acids) [41]. The larvicidal activities of fatty acids against larvae of *A*. *aegypti*, *A*. *albopictus* and *C*. *pipiens pallens* mosquitoes showed favourable results for mortality. However, the unsaturated fatty acids (oleic acid) were more responsible for toxicity against mosquito larvae as compared to saturated fatty acids (palmitic acid and tetradecanoic acid) [8]. Tetradecanoic acid was also proved by previous studies to result in larval mortality against *A*. *aegypti* and *C*. *quinquefasciatus* [26].

## Conclusion

Current study had shown the effectiveness of *A*. *catechu* nut extract against the fourth instar larvae of *Ae*. *aegypti* and *Ae*. *albopictus* mosquitoes with lower concentrations below 2268 mg/L. There are also no significant effects on *A*. *catechu* nut extracts for the non-target organism test. However, continuity of this research work is required to isolate the specific chemical compounds which may be responsible for its bio-insecticidal interactions. Additionally, the chemical components analysis from *A*. *catechu* nut extract had proven a baseline data for its future research on the field-based applications and their long-term effects on other aspects of human health.

## Supporting information

S1 File Chemical compounds from NIST 08 library search. (PDF)

S1 Table Basic chemical compounds information of *A*. *catechu* nut [33, 42–50]. (DOCX)

S1 Checklist *PLOS ONE* humane endpoints checklist. (DOCX)
