# Supplementary material for: Analysis of chemical compositions and larvicidal activity of nut extracts from Areca catechu Linn against Aedes (Diptera: Culicidae)
Source: PLoS One. 2021 Nov 29;16(11):e0260281. doi: 10.1371/journal.pone.0260281 (PMC8629262; doi:10.1371/journal.pone.0260281)

Sample Information

LIBRARY SEARCH
ID : 3
Sample Name : 3
Date :20.1.2020
Method :C:\GCMSsolution\Data\method\FFNSC method - Dr Rajiv.qgm

Library

<< Target >>
Line#:1 R.Time:12.265(Scan#:1754) MassPeaks:247
RawMode:Averaged 12.260-12.270(1753-1755) BasePeak:140.10(4867)
BG Mode:Calc. from Peak Group 1 - Event 1

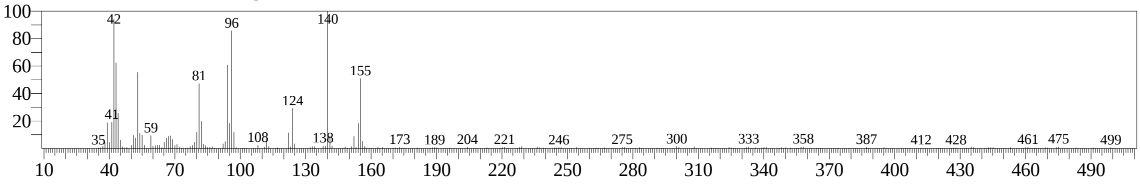

Hit#:1 Entry:9733 Library:NIST08s.LIB
SI:95 Formula:C8H13NO2 CAS:63-75-2 MolWeight:155 RetIndex:1171
CompName:3-Pyridinecarboxylic acid, 1,2,5,6-tetrahydro-1-methyl-, methyl ester $$ Nicotinic acid, 1,2,5,6-tetrahydro-1-methyl-, methyl ester $$ Are

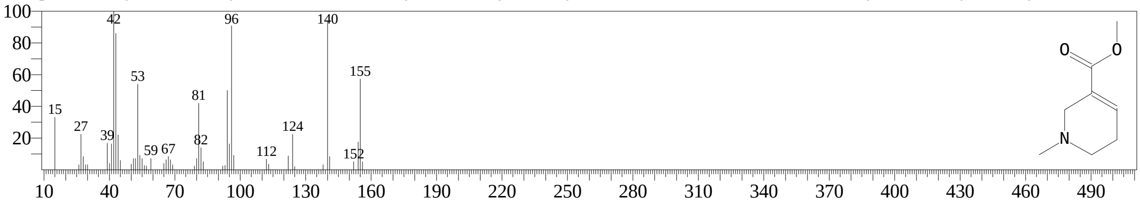

Hit#:2 Entry:10891 Library:NIST08.LIB
SI:77 Formula:C6H8N2O2 CAS:5962-13-0 MolWeight:140 RetIndex:1350
CompName:Urea, N-(2-furfuryl)- $$ N-(2-Furylmethyl)urea # $$

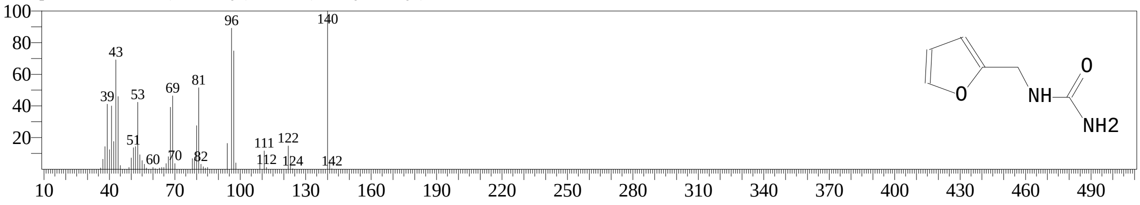

Hit#:3 Entry:11559 Library:NIST08.LIB
SI:75 Formula:C7H11NO2 CAS:499-04-7 MolWeight:141 RetIndex:1261
CompName:Arecaidine $$ Isoguvacine $$ Nicotinic acid, 1,2,5,6-tetrahydro-1-methyl- $$ 3-Pyridinecarboxylic acid, 1,2,5,6-tetrahydro-1-methyl- $$

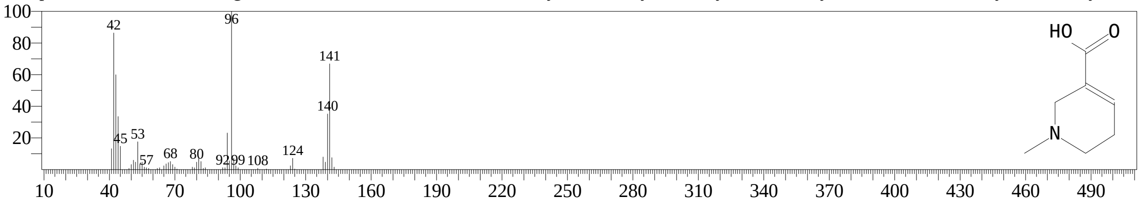

Hit#:4 Entry:10886 Library:NIST08.LIB
SI:73 Formula:C6H8N2O2 CAS:17759-08-9 MolWeight:140 RetIndex:0
CompName:Pyrimidine 1-oxide, 4-methoxy-6-methyl- $$ Methyl 6-methyl-1-oxido-4-pyrimidinyl ether # $$

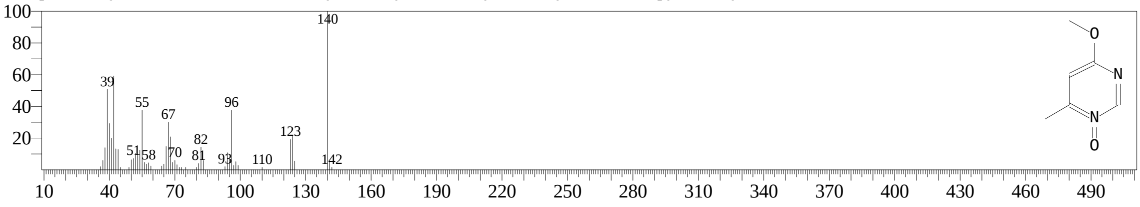

Hit#:5 Entry:59215 Library:NIST08.LIB
SI:72 Formula:C11H19N3O2 CAS:0-00-0 MolWeight:225 RetIndex:1573
CompName:5-Ethyl-6-imino-5-(1-methyl-butyl)-dihydro-pyrimidine-2,4-dione

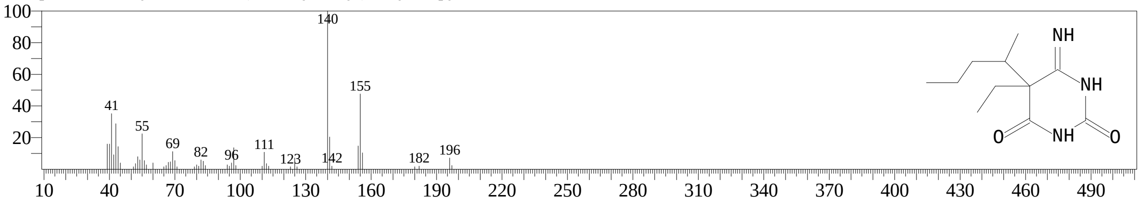

<< Target >>
Line#:2   R.Time:25.845(Scan#:4470) MassPeaks:242
RawMode:Averaged 25.840-25.850(4469-4471) BasePeak:73.00(1477)
BG Mode:Calc. from Peak Group 1 - Event 1

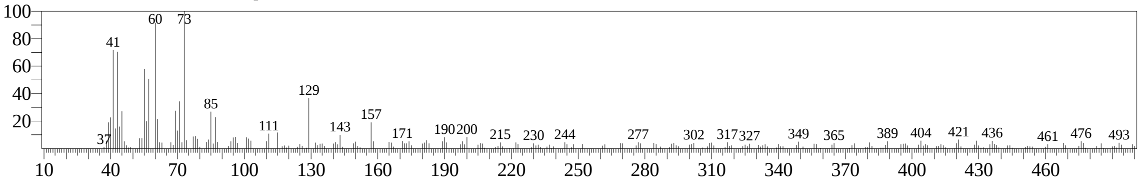

Hit#:1 Entry:16616 Library:NIST08s.LIB
SI:85 Formula:C12H24O2 CAS:143-07-7 MolWeight:200 RetIndex:1570
CompName:Dodecanoic acid $$ n-Dodecanoic acid $$ Neo-fat 12 $$ Aliphat no. 4 $$ Abl $$ Dodecylic acid $$ Lauric acid $$ Laurostearic acid $$ N

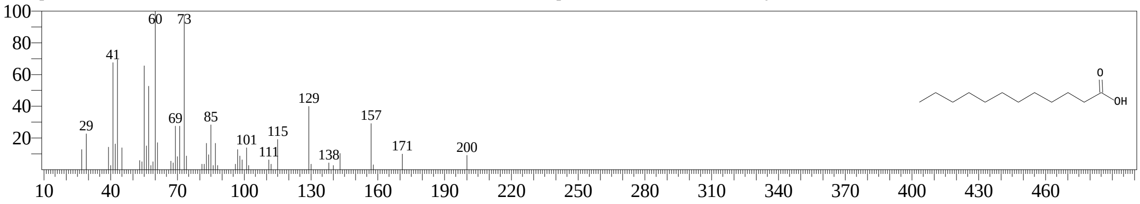

Hit#:2 Entry:14654 Library:NIST08s.LIB
SI:84 Formula:C11H22O2 CAS:112-37-8 MolWeight:186 RetIndex:1471
CompName:Undecanoic acid $$ n-Undecanoic acid $$ n-Undecoic acid $$ n-Undecylic acid $$ Hendecanoic acid $$ Undecylic acid $$ 1-Decanecarl

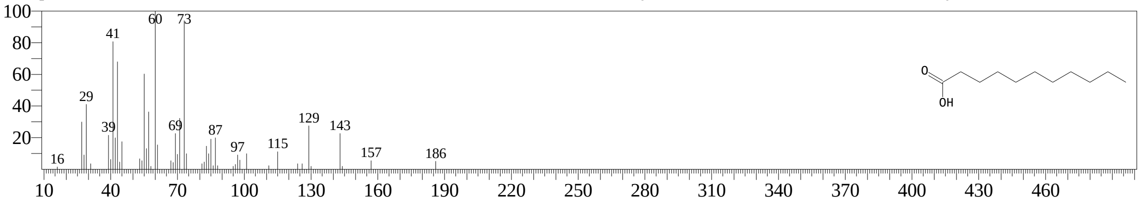

Hit#:3 Entry:12515 Library:NIST08s.LIB
SI:82 Formula:C10H20O2 CAS:334-48-5 MolWeight:172 RetIndex:1372
CompName:n-Decanoic acid $$ Decanoic acid $$ n-Capric acid $$ n-Decoic acid $$ n-Decylic acid $$ Capric acid $$ Caprinic acid $$ Caprynic acid

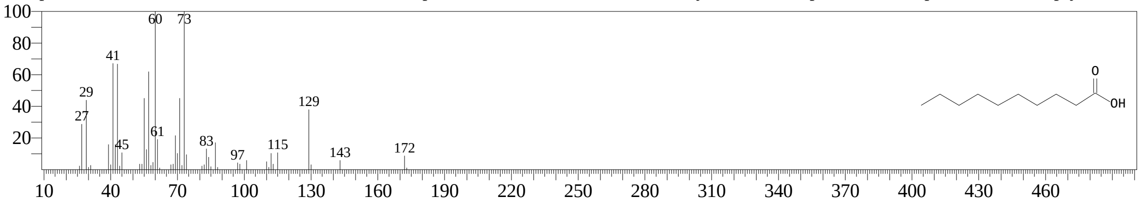

Hit#:4 Entry:52033 Library:NIST08.LIB
SI:82 Formula:C13H26O2 CAS:638-53-9 MolWeight:214 RetIndex:1670
CompName:Tridecanoic acid $$ n-Tridecanoic acid $$ n-Tridecoic acid $$ Tridecylic acid $$

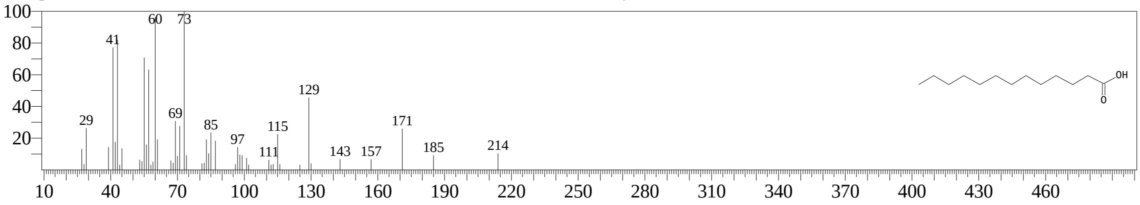

Hit#:5 Entry:20831 Library:NIST08s.LIB
SI:80 Formula:C15H30O2 CAS:1002-84-2 MolWeight:242 RetIndex:1869
CompName:Pentadecanoic acid $$ Pentadecylic acid $$ n-Pentadecanoic acid $$ n-Pentadecylic acid $$

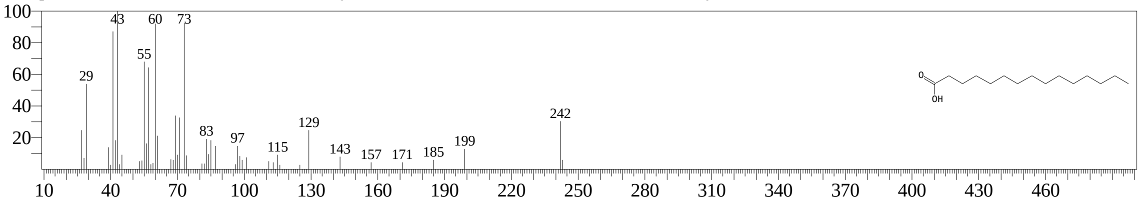

<< Target >>
Line#:3   R.Time:32.075(Scan#:5716) MassPeaks:281
RawMode:Averaged 32.070-32.080(5715-5717) BasePeak:74.05(4319)
BG Mode:Calc. from Peak Group 1 - Event 1

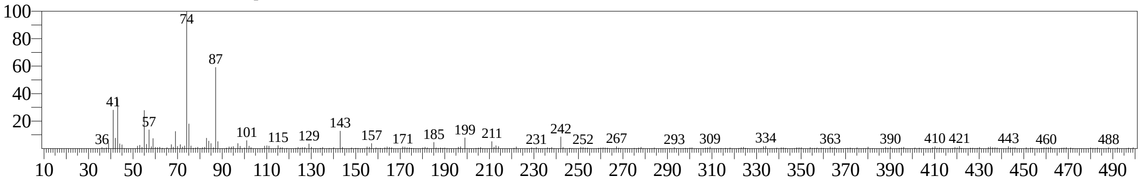

Hit#:1 Entry:20841 Library:NIST08s.LIB
SI:93 Formula:C15H30O2 CAS:124-10-7 MolWeight:242 RetIndex:1680
CompName:Methyl tetradecanoate $$ Tetradecanoic acid, methyl ester $$ Myristic acid, methyl ester $$ Metholeneat 2495 $$ Methyl myristate $$ M

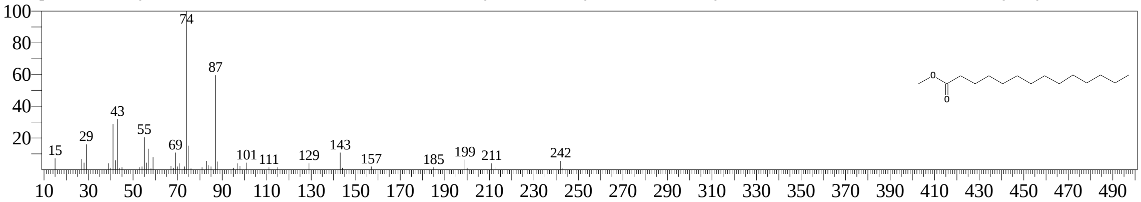

Hit#:2 Entry:100813 Library:NIST08.LIB
SI:91 Formula:C18H36O2 CAS:6929-04-0 MolWeight:284 RetIndex:1914
CompName:Hexadecanoic acid, 15-methyl-, methyl ester $$ Methyl isoheptadecanoate $$ Methyl 15-methylhexadecanoate $$

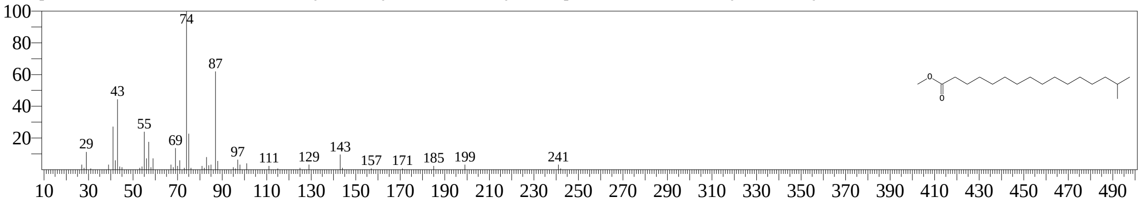

Hit#:3 Entry:70951 Library:NIST08.LIB
SI:91 Formula:C15H30O2 CAS:5129-58-8 MolWeight:242 RetIndex:1615
CompName:Tridecanoic acid, 12-methyl-, methyl ester $$ Methyl isomyristate $$ Methyl 12-methyltridecanoate $$

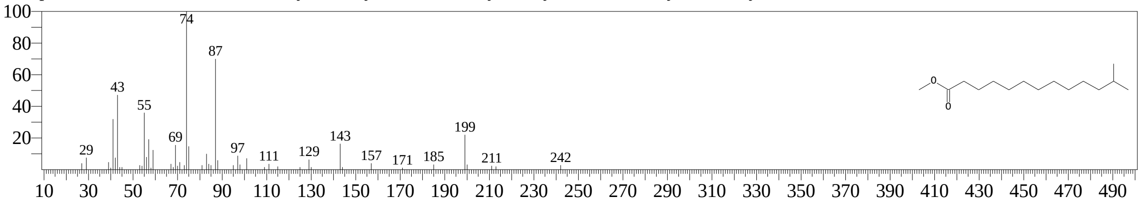

Hit#:4 Entry:90720 Library:NIST08.LIB
SI:90 Formula:C17H34O2 CAS:5129-60-2 MolWeight:270 RetIndex:1814
CompName:Pentadecanoic acid, 14-methyl-, methyl ester $$ Methyl 14-methylpentadecanoate # $$

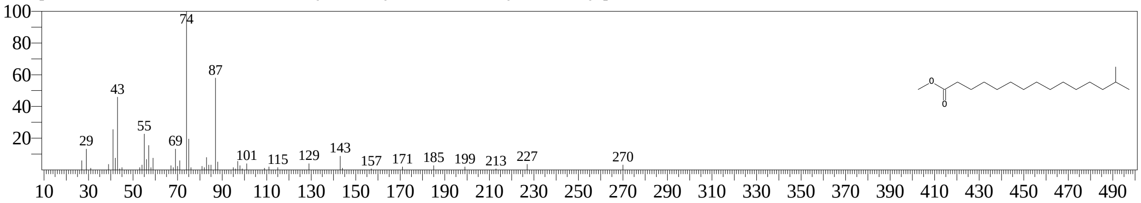

Hit#:5 Entry:24246 Library:NIST08s.LIB
SI:90 Formula:C19H38O2 CAS:112-61-8 MolWeight:298 RetIndex:2077
CompName:Octadecanoic acid, methyl ester $$ Stearic acid, methyl ester $$ n-Octadecanoic acid, methyl ester $$ Kemester 9718 $$ Methyl n-octade

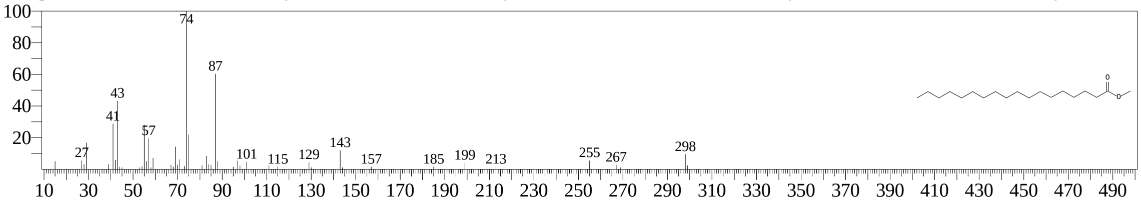

<< Target >>
Line#:4   R.Time:33.395(Scan#:5980) MassPeaks:269
RawMode:Averaged 33.390-33.400(5979-5981) BasePeak:43.05(2023)
BG Mode:Calc. from Peak Group 1 - Event 1

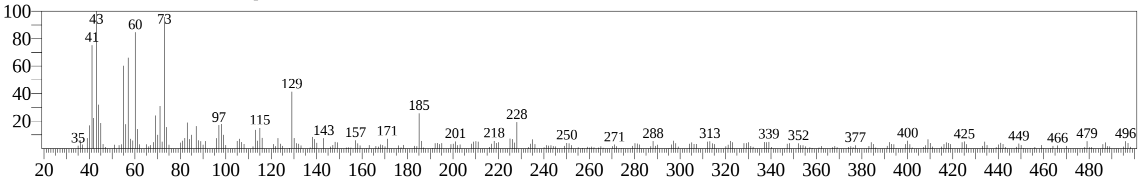

Hit#:1 Entry:950 Library:FFNSC1.3.lib
SI:86 Formula:C14 H28 O2 CAS:544-63-8 MolWeight:228 RetIndex:1773
CompName:Tetradecanoic acid <n->

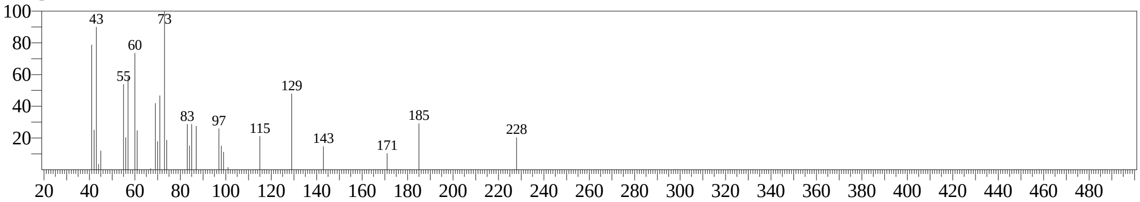

Hit#:2 Entry:52033 Library:NIST08.LIB
SI:86 Formula:C13H26O2 CAS:638-53-9 MolWeight:214 RetIndex:1670
CompName:Tridecanoic acid $$ n-Tridecanoic acid $$ n-Tridecoic acid $$ Tridecylic acid $$

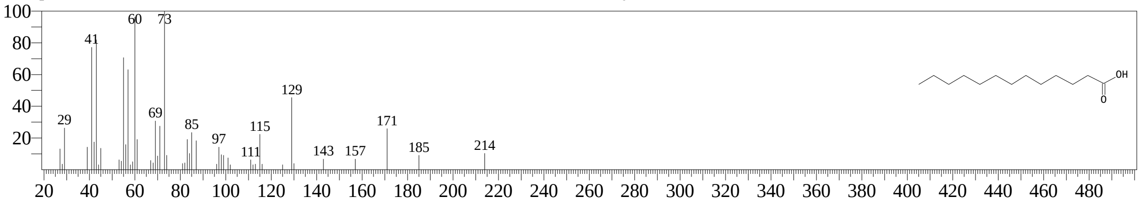

Hit#:3 Entry:20831 Library:NIST08s.LIB
SI:84 Formula:C15H30O2 CAS:1002-84-2 MolWeight:242 RetIndex:1869
CompName:Pentadecanoic acid $$ Pentadecylic acid $$ n-Pentadecanoic acid $$ n-Pentadecylic acid $$

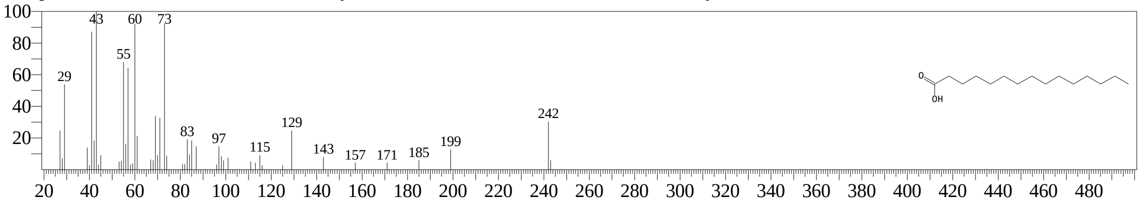

Hit#:4 Entry:23512 Library:NIST08s.LIB
SI:84 Formula:C18H36O2 CAS:57-11-4 MolWeight:284 RetIndex:2167
CompName:Octadecanoic acid $$ Stearic acid $$ n-Octadecanoic acid $$ Humko Industrene R $$ Hydrofol Acid 150 $$ Hystrene S-97 $$ Hystrene

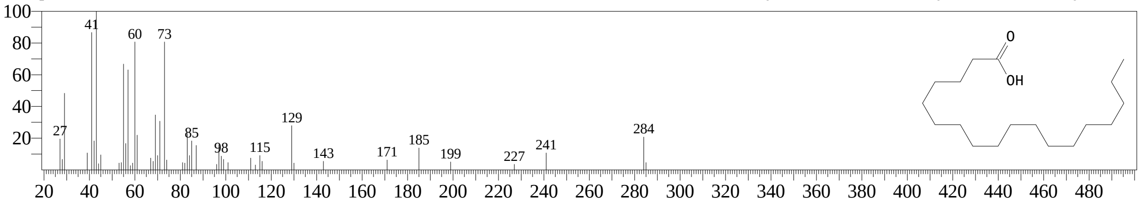

Hit#:5 Entry:80732 Library:NIST08.LIB
SI:83 Formula:C16H32O2 CAS:57-10-3 MolWeight:256 RetIndex:1968
CompName:n-Hexadecanoic acid $$ Hexadecanoic acid $$ n-Hexadecoic acid $$ Palmitic acid $$ Pentadecanecarboxylic acid $$ 1-Pentadecanecarbo

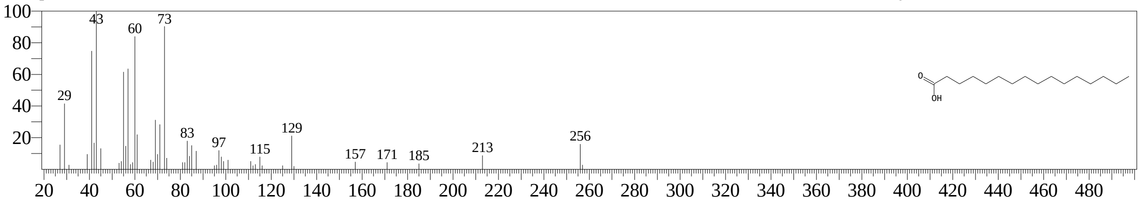

<< Target >>
Line#:5   R.Time:40.310(Scan#:7363) MassPeaks:247
RawMode:Averaged 40.305-40.315(7362-7364) BasePeak:43.05(1144)
BG Mode:Calc. from Peak Group 1 - Event 1

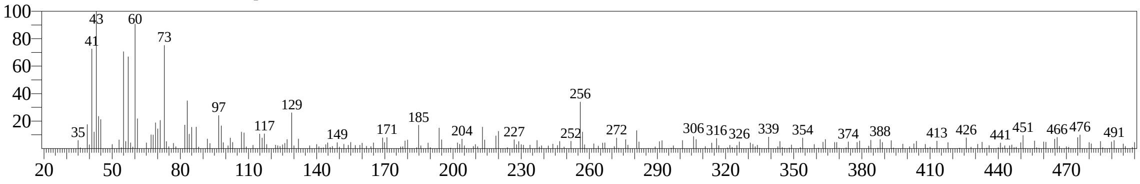

Hit#:1 Entry:21857 Library:NIST08s.LIB
SI:81 Formula:C16H32O2 CAS:57-10-3 MolWeight:256 RetIndex:1968
CompName:n-Hexadecanoic acid $$ Hexadecanoic acid $$ n-Hexadecoic acid $$ Palmitic acid $$ Pentadecanecarboxylic acid $$ 1-Pentadecanecarbo

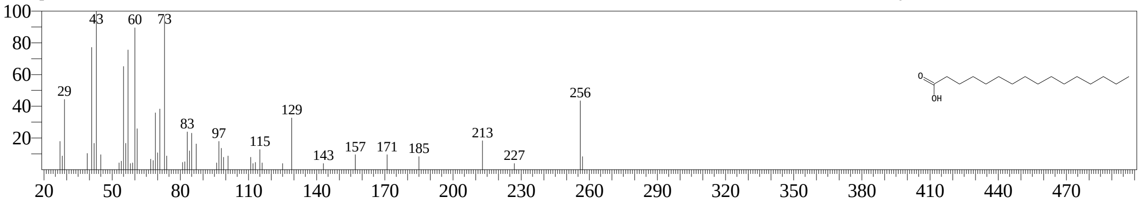

Hit#:2 Entry:23512 Library:NIST08s.LIB
SI:77 Formula:C18H36O2 CAS:57-11-4 MolWeight:284 RetIndex:2167
CompName:Octadecanoic acid $$ Stearic acid $$ n-Octadecanoic acid $$ Humko Industrene R $$ Hydrofol Acid 150 $$ Hystrene S-97 $$ Hystrene

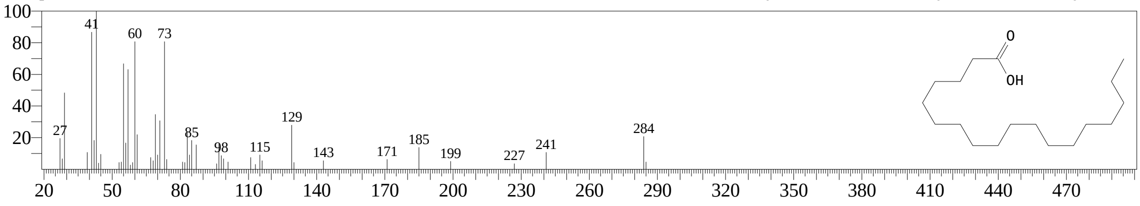

Hit#:3 Entry:52033 Library:NIST08.LIB
SI:77 Formula:C13H26O2 CAS:638-53-9 MolWeight:214 RetIndex:1670
CompName:Tridecanoic acid $$ n-Tridecanoic acid $$ n-Tridecoic acid $$ Tridecylic acid $$

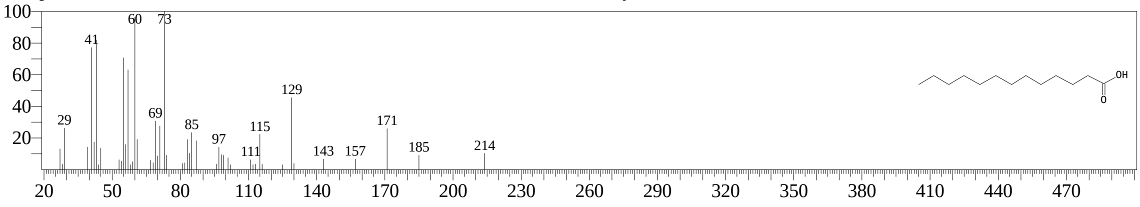

Hit#:4 Entry:120718 Library:NIST08.LIB
SI:77 Formula:C20H40O2 CAS:506-30-9 MolWeight:312 RetIndex:2366
CompName:Eicosanoic acid $$ Arachic acid $$ Arachidic acid $$ Icosanoic acid $$ n-Eicosanoic acid $$ Arachidic acid (synthetic) $$

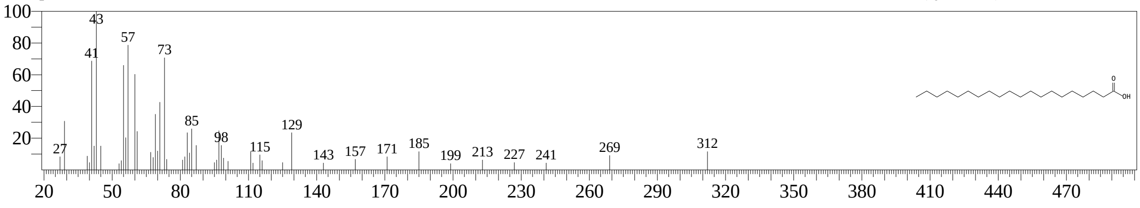

Hit#:5 Entry:20831 Library:NIST08s.LIB
SI:76 Formula:C15H30O2 CAS:1002-84-2 MolWeight:242 RetIndex:1869
CompName:Pentadecanoic acid $$ Pentadecylic acid $$ n-Pentadecanoic acid $$ n-Pentadecylic acid $$

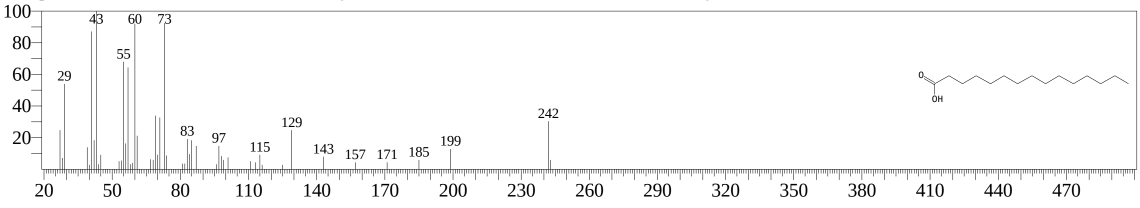

Supplement: S1 File — (PDF) [file pone.0260281.s001.pdf]
