# Supplementary material for: Analysis of chemical compositions and larvicidal activity of nut extracts from Areca catechu Linn against Aedes (Diptera: Culicidae)
Source: PLoS One. 2021 Nov 29;16(11):e0260281. doi: 10.1371/journal.pone.0260281 (PMC8629262; doi:10.1371/journal.pone.0260281)
Supplement: S1 Table — (DOCX) [file pone.0260281.s002.docx]

**S1 Table. Basic chemical compounds information of *A. catechu* nut.**

| Chemical compounds | Synonyms | Molecular formula | Molecular weight (g/mol) | 2-dimensional (2D) structure | Reference |
| --- | --- | --- | --- | --- | --- |
| Hexadecanoic acid methyl ester  Tetradecanoic acid  Oleic acid  9-octadecenoic acid (Z)- methyl ester  Palmitic acid | Methyl hexadecanoate,methyl palmitate, palmitic acid methyl ester  Myristic acid, n-tetradecanoic acid, n-tetradecoic acid  9-octadecenoic acid (Z)-, elaidoic acid, cis-9-octadecenoic acid  Methyl oleate, methyl 9-octadecenoate, oleic acid methyl ester  Hexadecanoic acid, n-hexadecanoic acid, pentadecanecarboxylic acid | C_17_H_34_O_2_  C_14_H_28_O_2_  C_18_H_34_O_2_  C_19_H_36_O_2_  C_16_H_32_O_2_ | 270.45  228.37  282.46  296.48  256.42 | 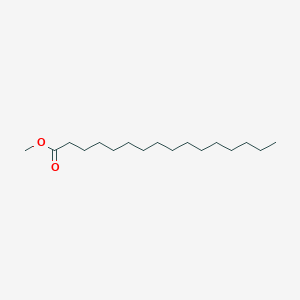  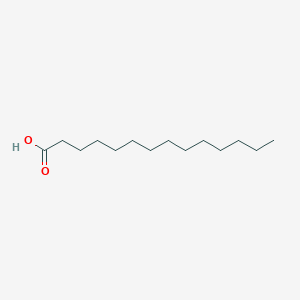  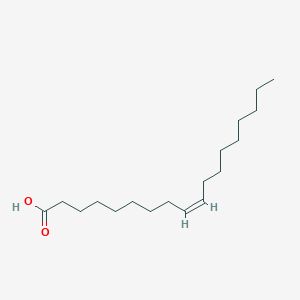  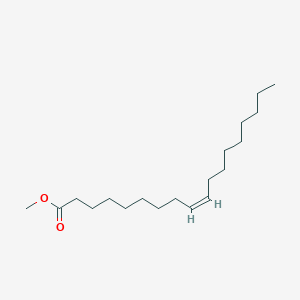  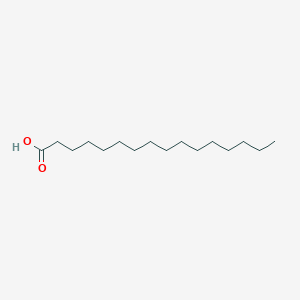 | [33, 42]  [43, 44]  [45, 46]  [47, 48]  [49, 50] |

**Table 3: Potential utilization of chemical compounds within *A. catechu* nut for pesticide formulations.**

| Patent Name | | Formulation Types | Chemical compounds | Patent Number |
| --- | --- | --- | --- | --- |
| Synergistic pesticide composition | | Adjuvant mixtures | Hexadecanoic acid methyl ester, oleic acid, palmitic acid, 9-octadecenoic acid (Z)-methyl ester, catechin | US9028856B2  [27] |
| Safer,organophosphorous compositions | | Adjuvant mixtures | Hexadecanoic acid methyl ester, oleic acid, palmitic acid, 9-octadecenoic acid (Z)-methyl ester, tetradecanoic acid | US6566349B1  [26] |
| Pest control using natural pest control agent blends | | Pesticide constituents | Oleic acid, palmitic acid, tetradecanoic acid. | US10368543B2  [29] |
| Pesticidal compositions and methods of use thereof | Repellent or cidal agents | Oleic acid | US9756857B2  [30] | |
